# Molecular mechanisms and biomarkers of total parenteral nutrition-induced hepatotoxicity revealed by iTRAQ proteomics analysis

**DOI:** 10.7150/ijms.122025

**Published:** 2025-10-10

**Authors:** Yung-Yu Hsieh, Jai-Jen Tsai, Shui-Yi Tung, Ko-Chao Lee, Kung-Chuan Cheng, Kam-Fai Lee, Meng-Chiao Hsieh, Cheng-Yi Huang, Chih-Chuan Teng, Chien-Heng Shen, Hsing-Chun Kuo

**Affiliations:** 1 Division of Gastroenterology and Hepatology, Department of Internal Medicine, Chang Gung Memorial Hospital, Chiayi 613016, Taiwan.; 2 College of Medicine, Chang Gung University, Taoyuan 333323, Taiwan.; 3 School of Medicine, National Yang Ming Chiao Tung University, Taipei, Taiwan.; 4 Taipei Veterans General Hospital, Yuan-Shan/Su-Ao Branch, Taipei City, Taiwan.; 5 Department of Nursing, Cardinal Tien Junior College of Healthcare and Management, New Taipei City, Taiwan.; 6 Division of Colorectal Surgery, Department of Surgery, Chang Gung Memorial Hospital-Kaohsiung Medical Center, Kaohsiung, Taiwan.; 7 College of Medicine, Chang Gung University, Kaohsiung, Taiwan.; 8 Department of Pathology, Chang Gung Memorial Hospital, Chiayi, Taiwan.; 9 Division of Colon and Rectal Surgery, Department of Surgery, Chang Gung Memorial Hospital, Chiayi, Taiwan.; 10 Department of Nursing, Division of Basic Medical Sciences, Chang Gung University of Science and Technology, Chiayi, Taiwan.; 11 Research Fellow, Chang Gung Memorial Hospital, Chiayi, Taiwan.; 12 Center for Drug Research and Development, Chang Gung University of Science and Technology, Taoyuan, Taiwan.; 13 Chronic Diseases and Health Promotion Research Center, Chang Gung University of Science and Technology, Chiayi, Taiwan.

**Keywords:** total parenteral nutrition (TPN), endoplasmic reticulum stress (ER stress), elongation of very long chain fatty acids protein 5 (Elovl5), polypyrimidine tract-binding protein 3 (Ptbp3), ASK1/mTOR/JNK/p38

## Abstract

**Background/Aims:** Total parenteral nutrition (TPN) provides medical nutrients intravenously to patients who cannot obtain proper nutrition through normal dietary means or enteral feeding. One significant concern is the risk of liver damage associated with long-term TPN use. In this study, the TPN-associated acute liver injury proteins and the molecular mechanisms underlying TPN oxidative stress were investigated through a quantitative proteomic survey. The proteomic changes between control and TPN infusion rats were analyzed by using the LC-MS/MS iTRAQ technology.

**Methods:** Rats were randomly assigned to saline infusion (control group) and TPN infusion (infusion rate of 30 mL/kg/h for 3 h). At the end of treatment, total liver samples from rats of control and TPN infusion groups were separated by iTRAQ-based quantitative proteomic identification. The effects of the differentially expressed proteins on the potential mechanism of hepatocytes were examined through flow cytometry. Additionally, siRNA-based assessments were conducted to examine the role of the endoplasmic reticulum stress (ER stress) as well as in vivo apoptosis of TPN-related liver cells.

**Results:** The effect of TPN on the biochemical markers of acute liver injury in the experimental rats was examined following palmitic acid treatment of live cells. Forty-eight proteins were differentially expressed between untreated control and TPN infusion liver tissues. The abundances of Elovl5 and Ptbp3 proteins were observed in TPN infusion (P < 0.05). Palmitic acid treatment of liver cells increased cell cytotoxicity and generated ROS, and increased the level of Elovl5 and Ptbp3, validated in the TPN infusion in vivo. The treatment of hepatocytes resulted in the activation of the caspases 3, caspase 9, accompanied by the expression and release of apoptotic molecules, cytochrome c, Bcl-2, Bcl-XL, p-IRE1α, and TRAF2. Elovl5 and Ptbp3 knockdown significantly regulated palmitic acid-mediated cytotoxicity of liver cells, including inhibition of apoptosis and ROS generation. Palmitic acid-mediated apoptotic induction was accompanied by histone H3K4 trimethylation of Elovl5 and Ptbp3 promoters, leading to enhanced transcription through the sustained phosphorylation of ASK1/JNK/p38 pathways.

**Conclusions:** The mechanism of palmitic acid-induced apoptosis cascade and ER stress in hepatocyte cells involves up-regulation of Elovl5 and Ptbp3. This study provides novel regulators underlying the effects of TPN on liver injury.

## Introduction

Total parenteral nutrition (TPN), alternatively referred to as intravenous or parenteral nutrition, represents a therapeutic modality implemented when a patient's gastrointestinal tract exhibits inadequate nutrient absorption capacity 1. For individuals, encompassing both pediatric and adult populations, who demonstrate insufficient ability to consume or absorb nutrients through conventional digestive processes, TPN assumes critical importance in sustaining nutritional adequacy 2,3. Prolonged reliance on TPN may precipitate hepatic injury, a notable complication manifesting as chronic cholestasis, steatosis, and cirrhosis. Additional terminology frequently employed to characterize these substantial complications includes intestinal atrophy, parenteral nutrition-associated liver disease, impaired glucose and lipid metabolism, cirrhosis, and hepatic failure; the absence of enteral feeding, recognized to disrupt normal enterohepatic circulation, potentially contributes to pathological conditions associated with TPN administration 3,4. The TPN is frequently associated with liver failure, caused by the pathophysiological process such as oxidative/nitrosative stress, characterized by increased hepatocytes apoptosis 5. The accumulation of reactive oxygen species (ROS) and peroxides in liver cells damages cellular components and causes cell injury, decreases liver oxidative metabolism capacity and enzymatic antioxidant defense 6,7. The TPN-related pathogenic mechanism and molecular targets for hepatitis are not clearly understood.

Several clinical and experimental studies demonstrate that TPN-induced oxidative stress is frequently associated with liver failure, damaged cellular components, and cell injury through mitochondrial dysfunction, frequently at a greater risk of TPN-mediated oxidative stress because of accumulation of ROS in liver cells 8. Oxidative stress affects the major cellular components, such as proteins, lipids, and DNA, and is charactrerized by upregulated activity of NADPH oxidase and decreased activity of cytosolic Cu-ZnSOD 9,10. An excess of oxidative agents contributes to lipid peroxidation and alters their function against peroxides and toxic compounds 11. Acute exposure to high levels of ROS may also cause serious damage within the body, such as during TPN infusion, which can lead to liver injury 12. In liver cells, ER stress disrupts lipid metabolism and causes mitochondrial dysfunction due to an excess of ROS, altering calcium homeostasis and protein misfolding, while saturated fatty acids induce ER stress and apoptosis via ASK1/mTOR/JNK/p38 pathways 13,14. Recent evidence showed that CHOP has a pro-apoptotic role in regulating hepatic metabolic genes during ER stress, activating multiple cellular signaling pathways. Among them, inositol-requiring enzyme type 1 (IRE1α) binds tumor necrosis factor (TNF) receptor-associated factor 2 (TRAF2), apoptosis signal-regulating kinase 1 (ASK1) and further activates Jun N-terminal kinase (JNK) 15, while its mechanisms of TPN-associated liver injury remains unclear.

TPN-associated proteins in the liver for early prognosis are treated as an urgent issue to identify reliable candidate markers 16. Among the identified TPN-associated proteins in rats, reliable candidate markers are yet to be developed. To examine the different functions related to expression profiles of various proteins associated with TPN involved in cytotoxicity, and ER stress in rats, proteomic analysis was conducted to process and identify differential protein profiles using iTRAQ-based (isobaric tags for relative and absolute quantitation) LC-MS/MS, followed by tissue microarray 17. In this study of liver injury of an acute TPN rat mode 18-21, we investigated whether experimental manipulation of expression of proteins, such as elongation of very long chain fatty acids protein 5 (Elovl5) and polypyrimidine tract-binding protein 3 (Ptbp3) can affect hepatotoxicity and the ER stress signaling pathway of the hepatocytes. We found intracellular signaling cascades involved in enhanced expression of Elovl5 and Ptbp3, including the production of ROS and ASK1/mTOR/JNK/p38 pathways. Further, these signaling pathways were found to affect a synergistic function in C/EBP Homologous Protein (CHOP) and transcription activation of the Elovl5 and Ptbp3 promoters. Cumulatively, of the roles of Elovl5 and Ptbp3 proteins as the novel biomarkers and molecular targets of TPN infusion, holds potential for early prognosis and treatment of TPN-related injury to liver cells.

## Materials and Methods

### Chemicals and reagents

Total parenteral nutrition solution Kabiven™ was acquired from Fresenius Kabi AB (Uppsala, Sweden). All culture materials were procured from Gibco (Grand Island, NY, USA). Primary antibodies targeting Bcl-2, Bcl-XL, cleaved caspase 3, cleaved caspase 9, cytochrome C, Elongation of very long chain fatty acids protein 5 (Elovl5) and Polypyrimidine tract-binding protein 3 (Ptbp3) and nitrotyrosine were obtained from Santa Cruz Biotechnology (Santa Cruz, CA, USA). The antibodies were respectively sourced from Cell Signaling Technology (Beverly, MA, USA) and Abcam Technology (Abcam, USA), including IRE1α (Ser724), TRFA2, JNK (Thr183), p38 (Thr180) and ASK (Thr1165). Protease inhibitor cocktails, antibody against β-actin, reactive oxygen species (ROS) scavenger (N-acetyl cysteine [NAC]), dihydroethidium (DHE), endoplasmic reticulum stress (ERS) inhibitor (TUDCA), p38 inhibitor (SB203580), JNK inhibitor (SP600125), 3-(4,5-dimethylthiazol-2-yl)-2,5-diphenyltetrazolium bromide (MTT), 2,7-dichlorodihydrofluorescein diacetate (H2DCFDA), SDS, NP-40, and sodium deoxycholate were purchased from Sigma (St. Louis, MO, USA). The TdT-mediated dUTP Nick End Labeling (TUNEL) kits were acquired from Roche (Germany).

### Animal experiments

Male Sprague-Dawley rats aged 6 weeks with body weights of 200 ± 25 g were acquired from Taiwan's National Laboratory Animal Center. These specific pathogen-free animals were provided unrestricted access to food (Laboratory Rodent Diet 5001, PMI Nutrition International LLC, MO, U.S.A.) and water and maintained under controlled environmental conditions with alternating 12-hour light and dark cycles. All experimental protocols involving these animals received review and approval from the Institutional Animal Care and Use Committee at Chang Gung Memorial Hospital, Chiayi, Animal Ethics Research Board (IACUC approval number: 2019032104). Isoflurane anesthesia was administered to the rats (5% for induction, 0.25-2.5% for maintenance). Each animal underwent catheterization of the left femoral vein to facilitate solution or TPN administration 17.

The experimental subjects (rats) received intravenous TPN infusion administered through the left femoral artery at a controlled flow rate of 30 mL/kg/h maintained for a duration of 3 hours to induce acute hepatic injury. For the control cohort, normal saline solution (0.9% sodium chloride) was employed as the vehicle. The SmofKabiven® formulation utilized provided a total energy content of 1000 kcal [comprising 13% glucose, 788 mL; amino acids with electrolytes, 456 mL; and lipid emulsion, 204 mL]. Each experimental animal received a cumulative infusion volume of 18 mL TPN administered at the specified rate of 30 mL/kg/h over the 3-hour intervention period. A representative rat weighing 200 g received an approximate energy delivery of 12.4 kcal during the procedure.

Male Sprague-Dawley rats (weighing 200-250g) were randomly assigned to either saline infusion (control group) or TPN infusion groups, with six animals per group. TPN was administered to all treatment animals through the left femoral vein for 3 hours at an infusion rate of 30 mL/kg/h. Blood samples were collected immediately through the left femoral vein and stored in heparin-coated capillary tubes for liver enzyme profiling and biochemical analysis. Liver tissue was harvested, washed with normal saline, and either stored at -80°C or fixed in 10% neutral buffered formalin for subsequent histopathological examinations 18.

### Histochemistry and immunohistochemistry

Immunohistochemistry stain (IHC) was performed using a biotinylated secondary antibody (Vectastain Universal Elite ABC Kit, Burlingame, CA, USA). Monoclonal rabbit antibodies against phospho-IRE1 alpha (Ser724), and phospho-ASK1 (Thr845) were diluted in a ratio of 1:200. The omission of primary antibodies was used as the negative control. For three slides, cytoplasm stained with brown was scored as positive. The expression of Protein abundances of Elongation of very long chain fatty acids protein 5 (Elovl5) and Polypyrimidine tract-binding protein 3 (Ptbp3) were quantitatively evaluated using Olympus Cx31 microscope with Image-pro Plus medical image analysis system. The digital images were captured using a digital camera (Canon A640). The positive area and optical density (OD) of Elovl5 and Ptbp3 positive cells were determined by measuring three randomly selected microscopic fields (400× magnification) for each slide. The IHC index was defined as average integral optical density (AIOD) (AIOD = positive area × OD/total area) 17-19. The TUNEL assay, initially designed as a standard histochemical method to detect DNA fragmentation resulting from apoptosis (TAAP01D, BioTna).

### Hepatocyte cell cultures and maintenance

The cell lines FL83B (CRL-2390) and HepG2 (HB-8065) were procured from American Type Culture Collection (ATCC). As delineated in a preceding publication, these cellular populations were maintained in F-12K Medium (Kaighn's Modification of Ham's F-12 Medium) and DMEM medium, respectively, with supplementation of 10% FBS (Gibco, USA) at a temperature of 37°C within an incubation environment containing 5% CO2.

### Apoptosis assay and oil red O stain in liver cells

The morphological characteristics of the cells stained with 4′ ,6-diamidino-2-phenylindole (DAPI) were observed under fluorescence microscopy. Firstly, the cells were fixed with 4% paraformaldehyde for 30 min at room temperature and were then permeabilized in 0.2% Triton X-100 in phosphate-buffered saline three times for 15 min. After PBS washing, the cells were incubated with 1 μg/mL of DAPI for 30 min. The percentage of the apoptotic nuclei in the field of the 200-300 cells was observed and scored. According to a previous report, a fluorescent microscope with a 340/380 nm excitation filter was used under 200 × magnification 22.

For Oil red O staining, the cells were fixed with 10% formalin for 30 min at room temperature followed by washing with PBS three times. After PBS was removed, 500 μl of 60% isopropanol was added and rinsed for 5 minutes to remove water. Following that, 1 mL of freshly prepared ORO solutions were added to the wells respectively and incubated for 15-20 minutes at room temperature. ORO solution was pipetted out, cells were washed with 500 μl of ddH_2_O for 15-20 seconds to remove excess dye solution, and then washed with PBS for more than 3 times until the liquid is clarified, pictures were captured (200×) under a microscope within 24 h.

### Measurement of lipid droplets and reactive oxygen species and mitochondrial membrane potential

The apoptotic cells (V+/PI-) were measured by the fluorescence-activated cell sorter analysis in a FACS analyser.^23^ Nile red: a selective fluorescent stain for intracellular lipid droplets and Nile red-stained, lipid droplet-filled cells exhibited greater fluorescence intensity than did nile red- stained control cells, and the cell populations could be analyzed by flow cytofluorometry. The intracellular accumulation of ROS (O2-) was determined using the fluorescent probes of DHE (Dihydroethidium) and the cells were washed prior to FACS analysis. JC-1 staining to examine the changes in mitochondrial membrane potential. The results are presented as a percentage of fluorescence intensity compared with the control sample. FACS analysis (Attune NxT Flow Cytometer, Thermo Fisher Scientific Inc., Waltham, MA USA). The data of the fluorescent intensity and the number of stained cells were quantified and analyzed and were represented as a percentage of the untreated control group in three independent experiments 24.

### Transfection with small Interfering RNA (siRNA)

Elovl5 siRNAs (Catalog: sc-62269), Ptbp3 (Catalog: sc-106897) and control siRNA (Catalog: sc-35448) were purchased from Santa Cruz Biotechnology (Santa Cruz, CA, USA). For transfection, cells were first incubated at 37°C overnight, p53 siRNA or control siRNA were transfected using Lipofectamine™ LTX Reagent with PLUS™ Reagent (Invitrogen™, Cat: 15538100), according to the manufacturer's instructions.

### Protein extraction, protein digestion, and iTRAQ labelling

Samples underwent rapid freezing in liquid nitrogen and subsequent lysis utilizing Lysis buffer (iNtRON PRO-PREP™). The extracted proteins were subjected to desalting procedures employing Amicon® Ultra-15 devices (Millipore) and subsequently quantified through implementation of the BCA assay (Thermo Fisher, USA). The resultant peptides were labeled with iTRAQ 4-plex reagents and solubilized in 0.5 M TEAB (pH 8.5). Reduction processes were conducted at 60°C for a duration of 30 minutes, followed by alkylation procedures performed at 37°C for an equivalent 30-minute period (utilizing TCEP in conjunction with iodoacetamide). Following concentration via SpeedVac drying, peptides underwent reconstitution and labeling in accordance with the protocol established by Applied Biosystems. Trypsin digestion was executed prior to the labeling process. Control group samples were designated with iTRAQ tags 114/115, whereas TPN group samples received tags 116/117, with each sample represented by two biological replicates 19.

### Database search and protein quantification

Raw MS data were analyzed using Mascot (v2.5) and Proteome Discoverer (v2.1) against the Swiss-Prot human protein database. Search parameters were previously described 19,25.

### Proteomic bioinformatic analysis

In iTRAQ analysis, differentially expressed proteins are selected based on a fold change >1.5 or <0.5, a P-value <0.05, and an FDR <0.05. Each protein must have at least one independent peptide identified, with a molecular weight >20 kDa and sufficient sequence coverage for reliable results. Protein annotation and pathway classification were performed using Gene Ontology (GO) and KOBAS 3.0 (KEGG). Statistically significant (FDR p < 0.05) differentially accumulated proteins were analyzed, with color intensity indicating significance. Protein-protein interaction networks were constructed using the STRING database (https://string-db.org) 19,25.

### Preparation of total cell extracts and immunoblot analyses

The cells were lysed with lysis buffer comprising sterile water, cell lysis solution (RIPA Buffer, Thermo Fisher, Waltham, MA, USA), (Sigma), and Protease inhibitor cocktails (Sigma). Proteins were separated on sodium dodecyl sulfate (SDS)-polyacrylamide gel by electrophoresis and transferred to the polyvinylidene fluoride (PVDF) membrane, and protein expression was detected using specific antibodies in the Chemiluminescence image system (Thermo Fisher Scientific, Waltham, MA, USA).

Quantitative analysis of the area of the photo images in the immunoblots for in terms of their numbers of pixels were performed using the ImageGauge 3.46 software (Fujifilm, Inc.) as previously described 26.

### Chromatin immunoprecipitation (ChIP) analysis

Cross-linked immunoprecipitated complexes were reversed at 65°C for 2 hours, followed by elution with a buffer containing 50 mM Tris-Cl (pH 7.5), 1 mM EDTA, and 1% SDS. DNA was purified using the ChIP DNA Clean & Concentrator Kit (Zymo Research). PCR was performed to amplify the promoter regions of **Ptbp3** and** Elovl5** under the following conditions: 94°C denaturation, 60°C annealing, and 72°C extension for 40 cycles. Specific primers were:

**Ptbp3 (-490 to -275 bp)** Forward: 5'-TGCTCTCAGGTCCGGAAAG-3', Reverse: 5'-CGACTCGCCCTCCTACAAG-3'.

**Elovl5 (-483 to -2181 bp)** Forward: 5'-TGCTTCAATTTGCGCAACAG-3', Reverse: 5'-AGTGAAGTTCTGGATGCCCT-3'. The percentage of input for each ChIP sample was calculated as **% Input = 2^(-ΔCt)**. 27,28

### Statistical analysis

Data are represented as mean ± standard deviation with three repeats from more than triplicate independent experiments. Using the SPSS software (version 10.0; SPSS, Chicago, IL, USA), statistically significant differences were evaluated with one-way ANOVA with post-hoc Mann-Whitney U test and Student's paired t-test and established at p < 0.05. 17,18,29.

## Results

### The effect of total parenteral nutrition on rat liver

**Figure 1:**
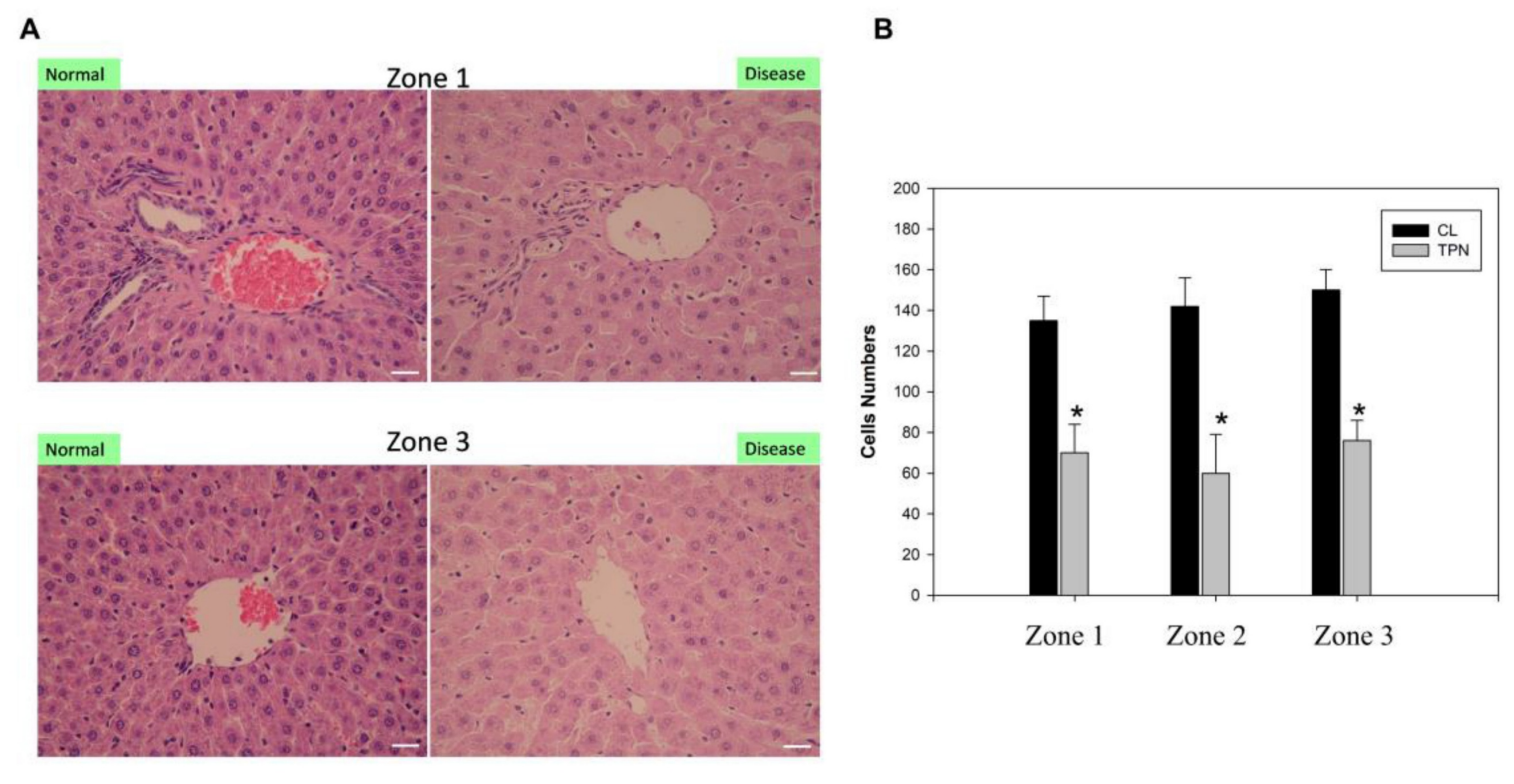
Histological examination of the total parenteralnutrition (TPN) rats. The livers were H&E-stained to examine the portal (Zone 1), mid-zonal (Zone 2) and terminal hepatic venules (Zone 3). (A) Saline infusion control (I); TPN infusion (II); staining of Zone 1 (left column) and Zone 3 (right column) of the liver. Liver pathology in the control group exhibited normal hepatocyte structure and arrangement. The TPN treated group exhibited damaged liver structure, as indicated by monocyte infiltration, steatosis and the presence of necrotic cells. (B) Quantitative evaluation of pathological examinations. Normal hepatocytes were counted from 10 random fields (200× magnification) of each liver sample and the values represent the mean ± SD of six rats.

Several studies have linked ER dysfunction and activation of the unfolded protein response (UPR), including IRE1, which is also positively correlated with the severity of liver disease 30. Our previous data demonstrated the generation of free radicals and ER stress induced by liver damage due to TPN 17,18. Based on these studies, we tested the differential protein expressions before and after oxidative liver damage and conducted molecular toxicology study to examine the generation of ER stress and hepatitis. For analyses, male Sprague-Dawley rats (weighing 200 to 250 g) were used. TPN was applied to all animals through the left femoral vein for 3 h at an infusion rate of 30 mL/kg/h 17,18. The rat livers of the TPN group exhibited obvious inflammatory reactions and hepatocyte death in the portal zone Z1, periportal zone Z2, and terminal hepatic venule Z3. Compared to the control group, there was a significant decline in the number of hepatocytes of the TPN group (Figure 1). The quantitative analysis showed that the number of hepatocytes in the Z1, Z2, and Z3 zones of the control group was 135 ± 8, 140 ± 10, and 145 ± 11 respectively, while that of the TPN group was 68 ± 5, 60 ± 8, and 70 ± 8 (p<0.05) respectively. There were no significant differences of livers appearance were observed between the two groups in liver size or weight during tissue collection from the rats. Further, the serological examination showed a significant increase in the liver damage index of rats of the TPN group and the levels of ALT, AST, and ALP were, respectively, 139 ± 5, 84 ± 3 and 641 ± 7, while that of the control group were 40 ± 5, 25 ± 4, and 544 ± 8 (p<0.05), respectively (Table 1). These results indicate that the TPN-induced liver injury is accompanied by significant abnormalities in the liver function and histopathological changes. In future research, we will focus on understanding the molecular mechanisms of these pathological changes and explore related protein markers for better understanding of the mechanism of TPN-induced liver injury.

### Analysis of differentially expressed proteins in hepatocytes from rats following total parenteral nutrition-induced liver injury by iTRAQ-based quantitative proteomics

**Figure 2:**
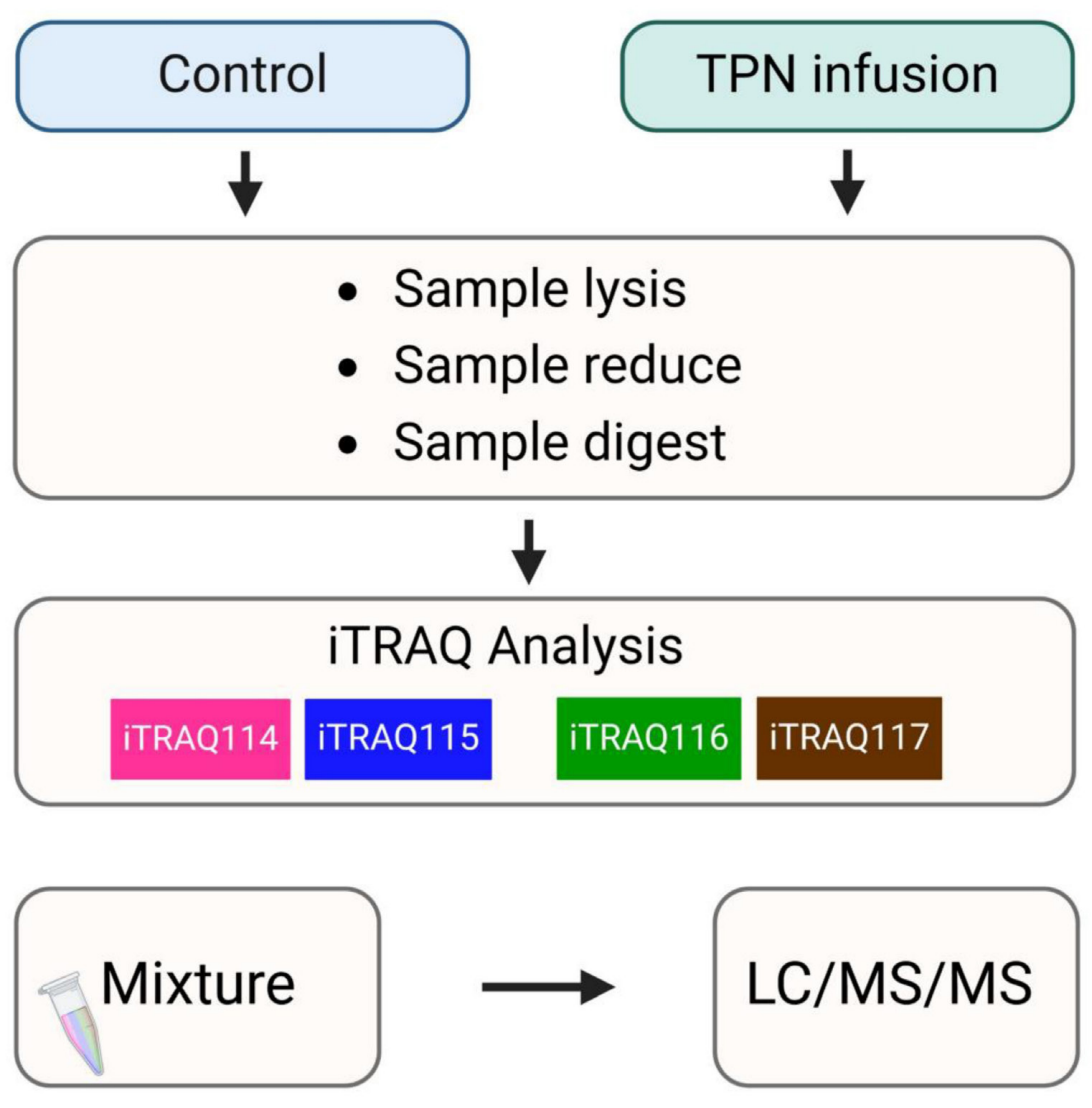
Schematic flowchart of the iTRAQ method by TPN rat model.

**Figure 3:**
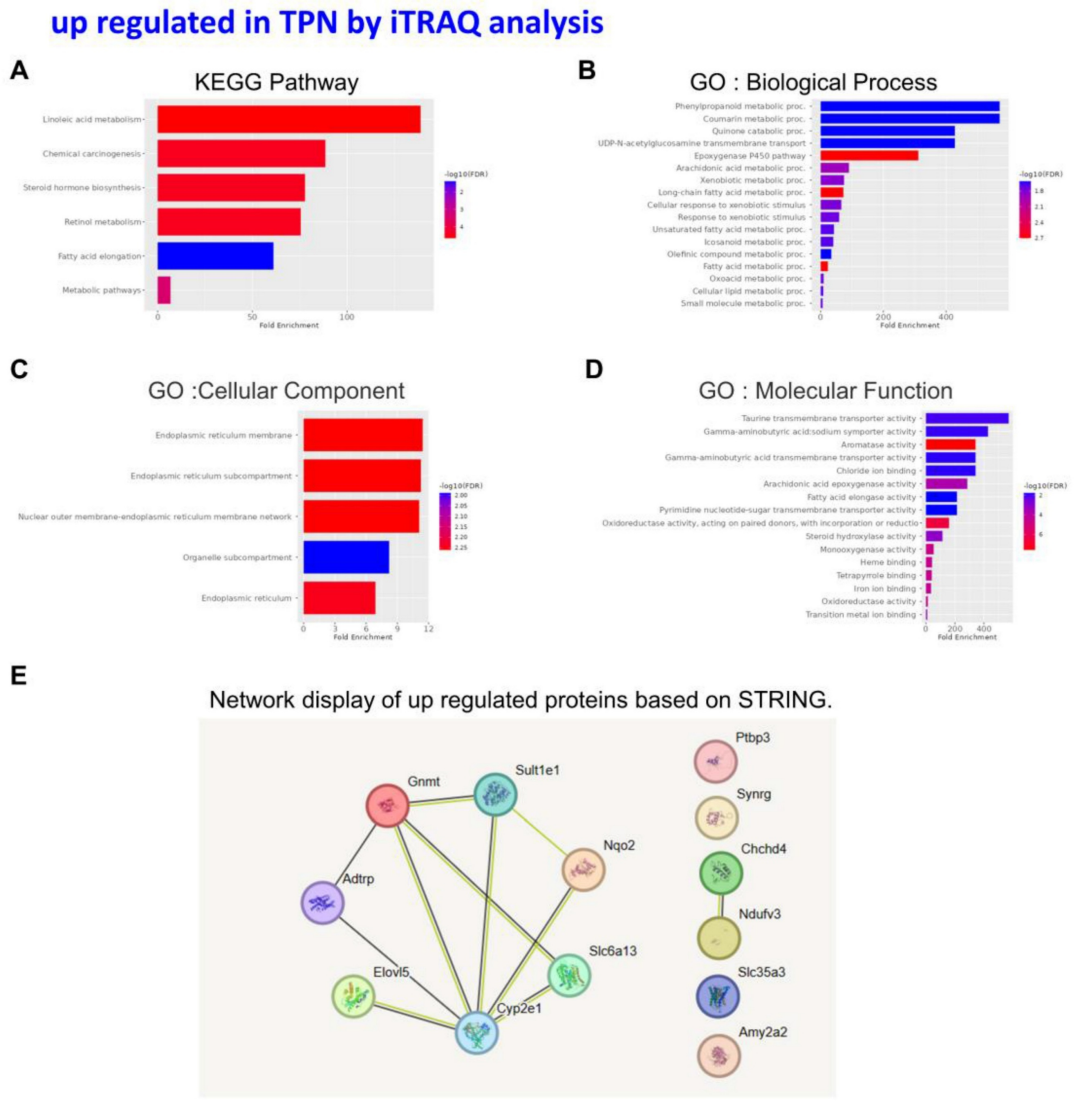
Classification of the identified proteins upregulated from rats following TPN-treatment by the KEGG, GO and STRING database. KEGG pathway (A) Biological process (B) Cellular component (C) Molecular function (D) STRING (E).

**Figure 4:**
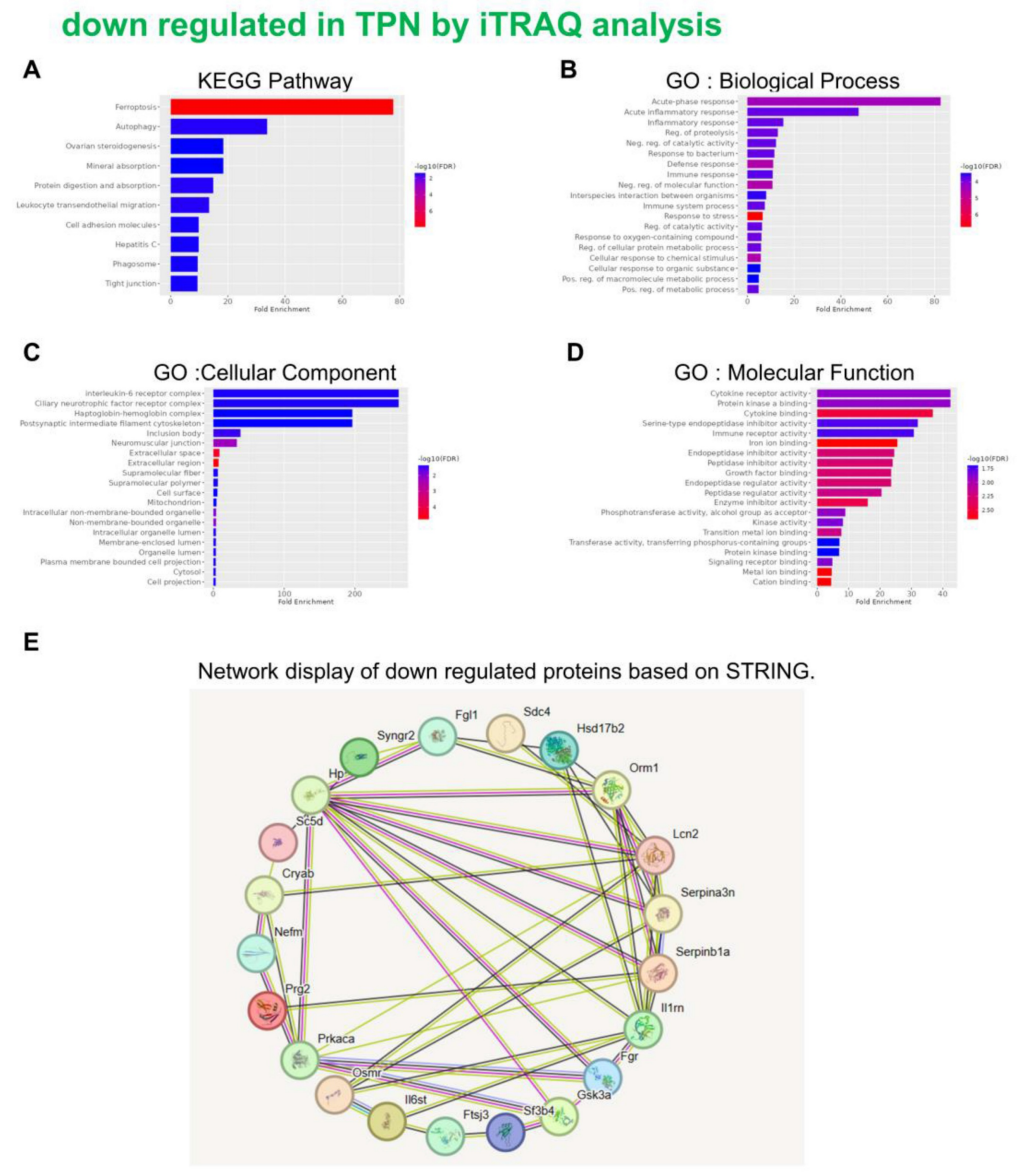
Classification of the identified proteins downregulated from rats following TPN-treatment by the KEGG, GO and STRING database. KEGG pathway (A) Biological process (B) Cellular component (C) Molecular function (D) STRING (E).

An in-depth proteomic analysis of TPN-induced liver injury was conducted, aiming to identify the biomarkers of liver inflammation and explore the associated mechanism. Liver tissues were collected from the animals of various groups, and isotope labeling technique was used to label the proteins in liver tissues to explore TPN-induced liver inflammatory reaction. The iTRAQ chemical calibration method was combined with mass spectrometry to analyze the tissue samples of TPN-induced liver injury, to evaluate the changes in relative protein content (Figure 2). A total of 6,436 proteins were identified in this study, of which quantitative results of 48 proteins were obtained. The analysis further revealed 19 proteins with significantly increased expression (Table 2) and 29 proteins with significantly reduced expression (Table 3) in the control group and TPN group (difference exceeding 1.5 times). Screening and classification of these differentially expressed proteins, Elovl5 and Ptbp3, revealed enhanced expression in the TPN group, and the important role of these proteins in lipid metabolism was shown. These 19 differentially expressed proteins were classified using the PANTHER (Protein Analysis Through Evolutionary Relationships) Classification System (www.pantherdb.org). Further analysis of their molecular and functional characteristics revealed in 6 Kyoto Encyclopedia of Genes and Genomes (KEGG) pathways, 17 biological processes, 5 cellular components, 16 molecular functions, and their network displays were classified (Figure 3). Figure 3 shows a 19-protein network of interactions by using the String database (http://string-db.org), a helpful tool for eliminating protein groups involved in a particular pathway 19,25. The protein interaction network diagram generated by the String database further revealed the interaction between Elovl5 and Ptbp3 proteins in lipid metabolism. These results showed that the expression levels of Elovl5 and Ptbp3 are closely related to ER stress and lipid metabolism. Further, Elovl5 affects the composition of cellular lipid pools by extending fatty acids 31, while Ptbp3 possibly participates in the ER stress response by regulating the UPR pathway, to reduce the ER stress 32,33. We also extensively analyzed 29 proteins with reduced expression in the TPN group, and employed the PANTHER system for classification, covering 10 KEGG pathways, 19 biological processes, 20 cellular components, and 20 molecular functions (Figure 4). The results showed that in TPN-induced liver injury process, the functions and interaction network of these proteins may play an important role in the pathological changes in the liver. Noteworthily, more specific information regarding Elovl5 and Ptbp3 protein abundances and functional characteristics in hepatocytes from rats following TPN-induced liver injury may be determined, particularly in the context of fatty acid metabolism and ER stress, through further experimental studies such as proteomic analyses, gene expression profiling, and functional assays.

### Validation of the expression patterns of Elovl5 and Ptbp3 validated *in vivo* in the TPN infusion

**Figure 5:**
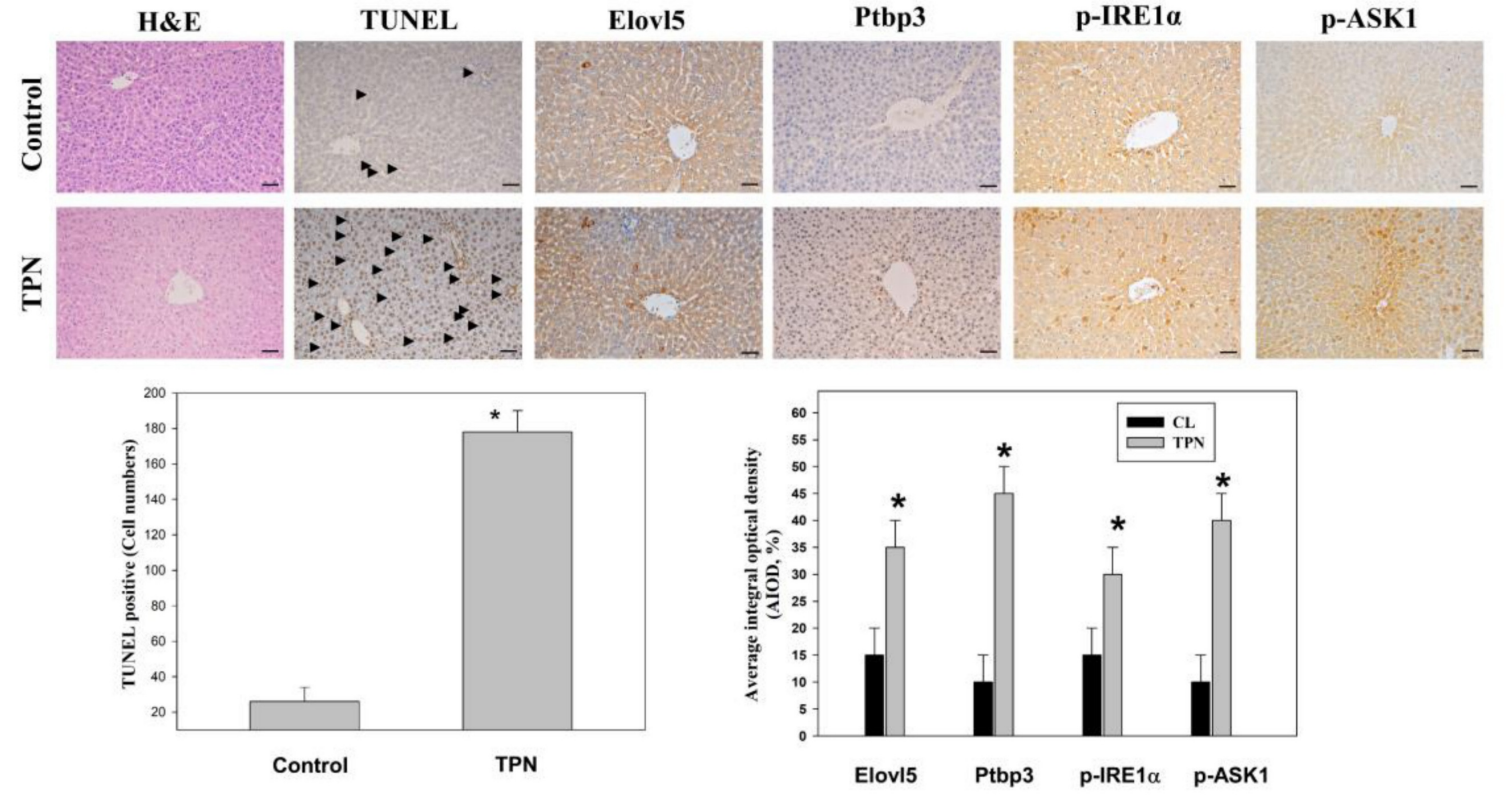
Effects of TPN infusion-induced liver tissue damage in rats' livers from terminal hepatic venules (Zone 3). (A) Rats were randomly administered to saline infusion as control group (Control, n = 6) and TPN infusion (n = 6, an infusion rate of 30 mL/kg/hr for for 3 hours and were determined for liver tissue. Representative liver sections of hematoxylin and eosin (H&E) stained with control group and TPN group animals. Immunohistochemical analysis of these livers from each treatment group was conducted and evaluated in multiple tissue fields. Representative IHC (for TUNEL, ELOVL5, Ptbp3, ASK1 (phospho Thr845) and IRE1α (phospho Ser726) proteins) and H&E staining images in each treatment group are presented. Quantitative immunohistochemical were evaluated with average integrated optical density (AIOD). The positive stained area was evaluated from three randomly selected observation fields of each tissue section. Data are expressed as mean ± S.D. (n = 6 per group). *p < 0.05, compared with the control group, magnification ×200.

An immunostaining analysis system was used to observe the changes in liver tissue through H&E staining and immunohistochemical staining techniques 25. These analyses included antigen processing, blocking of endogenous catalase, application of the primary antibody and the secondary antibody, and sealing after DAB staining. The expression of specific proteins in the liver was examined. The proteins of special concern included Elovl5, Ptbp3, phospho-IRE1 alpha (Ser724), and phospho-ASK1 (Thr845). Immunostaining analysis revealed that the expression levels of Elovl5 and Ptbp3 were significantly higher, by 2-4 times, in the TPN-induced liver injury group than the control group. In addition, the two ER stress-related proteins, phospho-IRE1 alpha (Ser724) and phospho-ASK1 (Thr845), had significantly increased expression in the TPN group, the difference also being 2-4 times (Figure 5). The death of liver cells in the TPN group was verified by terminal deoxynucleotidyl transferase dUTP nick end labeling (TUNEL) staining. The death rate of liver cells in the terminal hepatic venule Z3 zone was five times that of the control group (Figure 5). These results further prove the effect of the TPN-induced ER stress on liver injury and emphasize the importance of Elovl5, Ptbp3, and other key proteins in the pathological changes in the liver.

### Effects of differentially expressed proteins, Elovl5 and Ptbp3 on the lipotoxic effect of palmitate acid on liver cells

**Figure 6:**
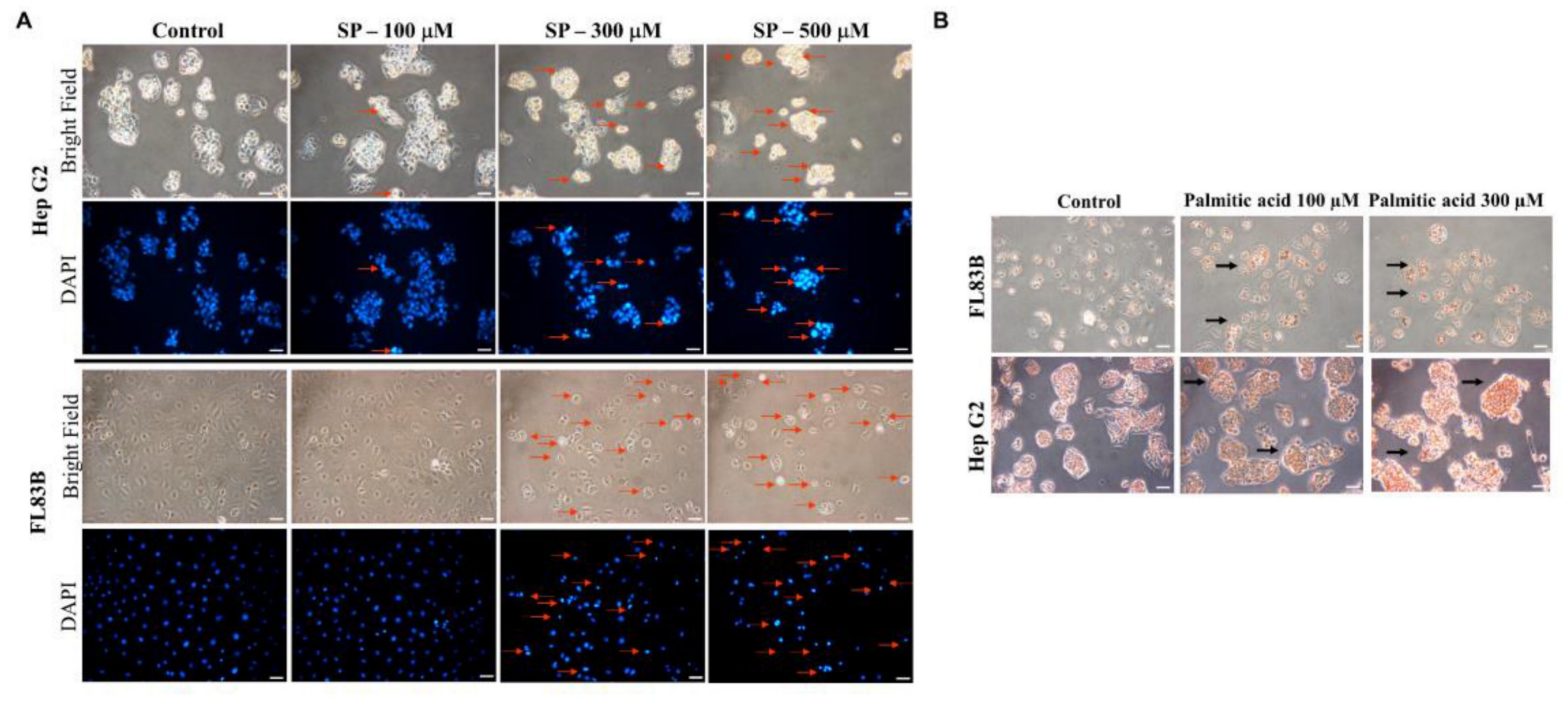
Changes in nuclei by DAPI staining and Oil Red O. Liver cells were treated with Palmitate, and stained with DAPI. (A) Apoptotic cells were measured under fluorescence microscopy as described. Apoptotic cells indicate condensed and damaged nuclei (red arrows). Magnification × 200. (B) Representative bright-field images of Oil Red O stained cells.

**Figure 7:**
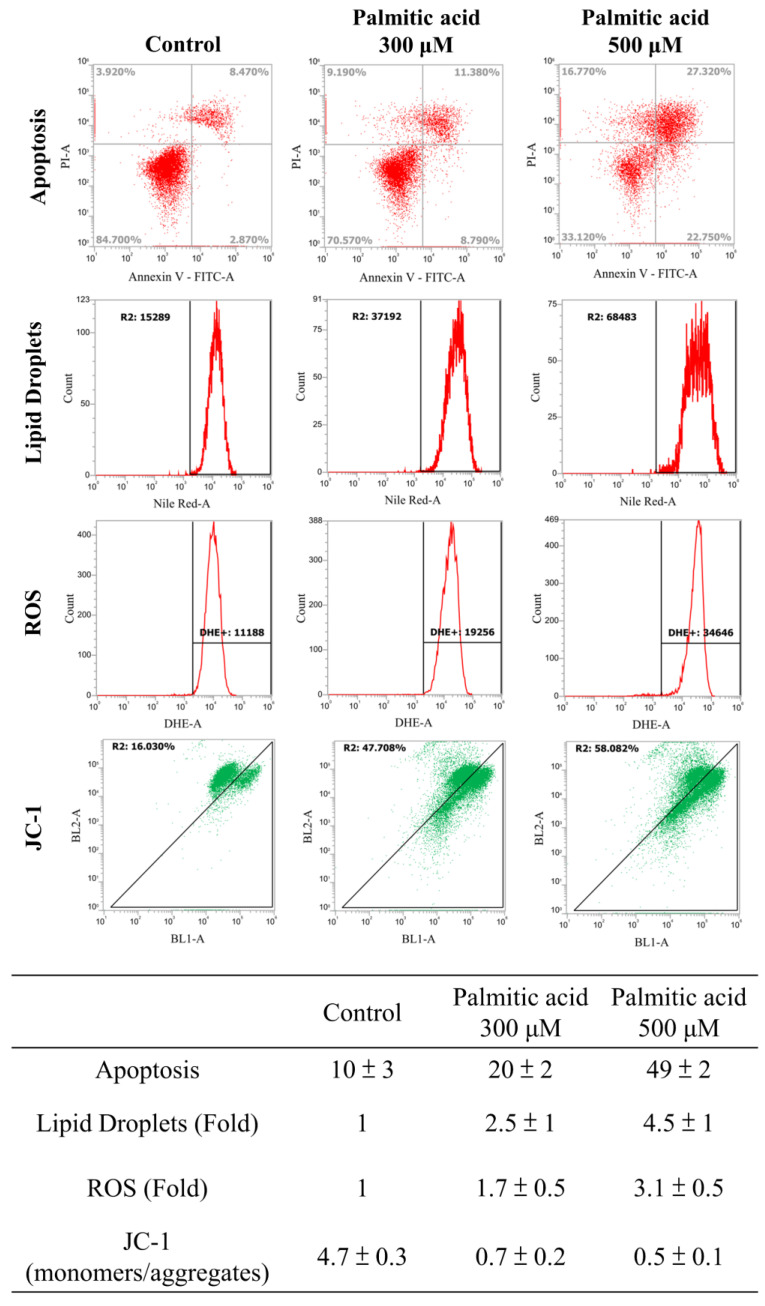
Cells were treated with Palmitate, and induces cell apoptosis, increased lipid accumulation and ROS production and decreasing mitochondria potential in liver cells. After 24 hours of palmitic acid treatment, the hepatocytes were stained with FITC-conjugated Annexin-V and PI for a flow cytometry analysis, as described in the Materials and Methods section. After receiving individual treatment, the percentage of the apoptotic cells is shown in each frame, as indicated. Nile red staining of liver cells and quantitative analysis by FACS. Fluorescence dot plot and relative fluorescence graph. The intracellular ROS of cells treated with palmitic acid for 24 hours was measured by using a FACS analysis, as described in the Materials and Methods section. Representative histograms of typical H2DCFDA/Fluo-3 profiles are shown. The production of ROS was expressed as the fold of the control group. The membrane-permeant JC-1 dye exhibits potential-dependent accumulation in mitochondria, indicated by palmitic acid, which is indicated by a decrease in the green/red fluorescence intensity ratio. Data are presented, based on three independent experiments, as mean ± S.D.

Multiple experimental methods were used to observe and analyze the death of cell nuclei, fat vacuole formation, cell viability, lipid oil droplet formation, ROS generation, and mitochondrial membrane potential changes. First, significant changes in the nuclear death of hepatocytes induced by different doses of palmitate acid (0, 100, 300, 500 μM) were observed by DAPI staining and fluorescence microscopy, that revealed significantly increased cytotoxicity with the increase in palmitate concentration (Figure 6A). Next, the Oil-Red-O stain revealed that the palmitate induced the formation of fat vacuoles in hepatocytes, showing a dose-dependent effect of palmitate on lipid accumulation (Figure 6B). The results of Annexin-V FITC/PI fluorescence staining to measure the apoptosis rate of cells showed that the apoptosis rates of hepatocytes were 10%, 20%, and 49%, upon the use of different concentrations of palmitate (0, 100, 300 μM), suggesting a significant apoptosis-inducing effect on hepatocytes (Figure 7). In addition, Nile Red staining was performed and detected through flow cytometry. After 24 h of palmitate treatment, the production of lipid oil droplets in hepatocytes increased significantly, with a significant increase of 1, 2.5, and 4.5 folds, respectively, as the concentration increased (Figure 7). The changes in intracellular ROS production were quantified using DHE staining; with the increase in palmitate concentration (0, 300, 500 μM), the ROS production in the hepatocytes increased significantly, reaching 1, 1.7, and 3.1 fold respectively (Figure 7). In addition, JC-1 staining to examine the changes in mitochondrial membrane potential showed that after 24 h of palmitate treatment, the mitochondrial membrane potential of hepatocytes was significantly reduced, with corresponding rates of 4.7, 0.7, and 0.5, demonstrating a serious damage to the mitochondrial function (Figure 7). These experimental results indicate a significant toxic effect of palmitate acid on hepatocytes, including apoptosis induction, promoting lipid accumulation, increasing ROS generation, and damaging mitochondrial function.

### Effects of palmitate acid on protein expression and signaling pathways in hepatocytes

**Figure 8:**
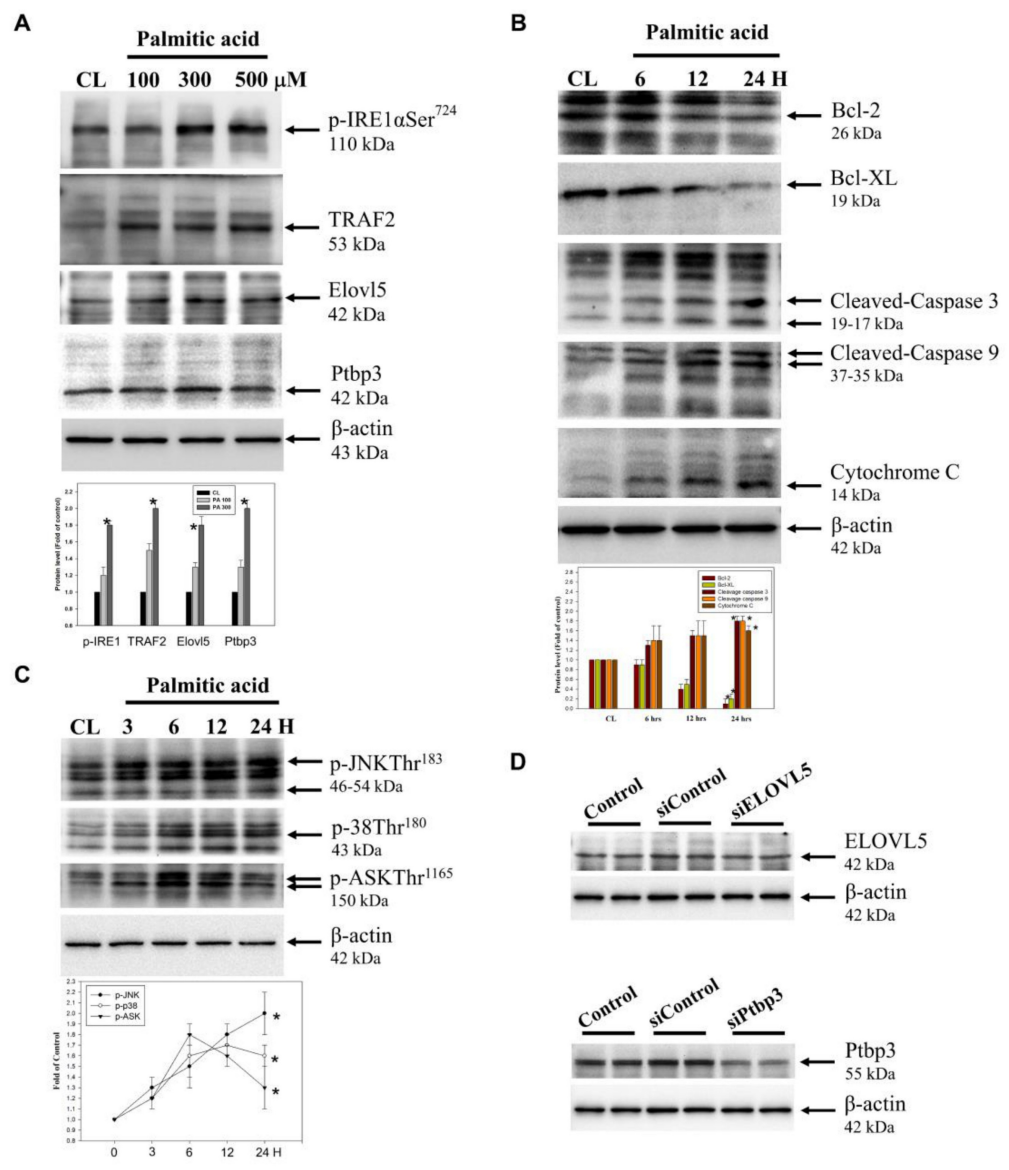
The effect of Palmitate on ER stress-related proteins, hepatocyte injury and and ASK/JNK/p38 MAPK pathways in liver cells. (A, B, C) The protein levels of p-IRE1, TRAF2 and Elovl5 and Ptbp3 ae well as Bcl-2, Bcl-XL, caspase-3, caspase-9, cytoplasmic Cytochrome C, phosphorylation of ASK, JNK, and p38 were determined using Western blotting in sodium palmitate-treated cells. While β-actin served as an internal control, densitometric analysis was used to quantify protein levels with the control at 100%. Data are expressed as the mean ± SD of three independent experiments. * p < 0.05, compared with the control group. (D) Protein lysates of the cells from control, siControl, siElovl5, and siPtbp3 clones were subjected to western blot analysis.

In cell model, palmitate acid induces lipotoxicity, leading to ROS accumulation and subsequent activation of these signaling pathways. The combined activation of JNK, p38, and ASK1 and aggravates ER stress and promotes apoptotic cell death, contributing to the overall lipotoxic effects of palmitate acid 7,8. The expression of proteins was tested using western blotting. The free radicals are generated through the ER stress signaling pathway and the process involves two types of proteins: Ire1α/TRAF2. The effects of the ER stress on the differentially expressed proteins Elovl5 and Ptbp3 during hepatitis were further demonstrated. When the palmitate acid concentration was: 0, 100, 300, 500 μM, the treatment of hepatocytes for about 24 h, led to the expression of p-Ire1α/TRAF2, Elovl5, and Ptbp3 (Figure 8A). B-cell lymphoma protein 2 (Bcl-2) is a critical protein in the cell apoptosis. In mitochondria, the Bc1-2 family protein interacts with other apoptins to regulate the stability of mitochondrial structure and function, and acts as the "main switch" for cell apoptosis. The Bcl-2 family includes two kinds of proteins: the anti-apoptotic protein Bcl-2/Bcl-XL, and the pro-apoptotic protein Bcl-2-associated X (Bax). When hepatocytes were treated with palmitate acid at 500 μM for 24 h, there was a decrease in the expression of Bcl-2/Bcl-XL and the release of activated caspase-3/-9 and cytochrome C to the cytoplasmic (Figure 8B). Moreover, this treatment also activated the ROS/ASK/JNK/p38MAPK pathway, especially within the 3-24 h of palmitate acid induction (Figure 8C). Thus, the treatment of hepatocytes led to the induced of the ROS/ASK/JNK/p38MAPK pathway. Therefore, to determine the potential mechanisms of Elovl5- and Ptbp3-mediated cell apoptosis and lipid droplet generation, we transfected siRNAs for Elovl5 and Ptbp3 or scrambled siRNA control particles into hepatocytes and consequently observed downregulated levels of Elovl5 and Ptbp3 proteins (Figure 8D). As shown in Table 5, siRNAs for Elovl5 and Ptbp3 blocked palmitate treatment-induced cell death by 20%, 13%, and 11%, respectively. The siRNA-mediated blockage of Elovl5 and Ptbp3 expression reduced palmitate-induced ROS generation almost by 1.7, 1.2, and 1.3 fold. The elevated levels of Elovl5 and Ptbp3 demonstrate the impact of palmitate-induced ER stress on liver injury and highlight the critical roles of Elovl5, Ptbp3 in the pathological changes observed in liver cells.

### Effect of palmitate acid on gene expression of Elovl5/Ptbp3 and its correlation with H3K4me3 histone modifications

**Figure 9:**
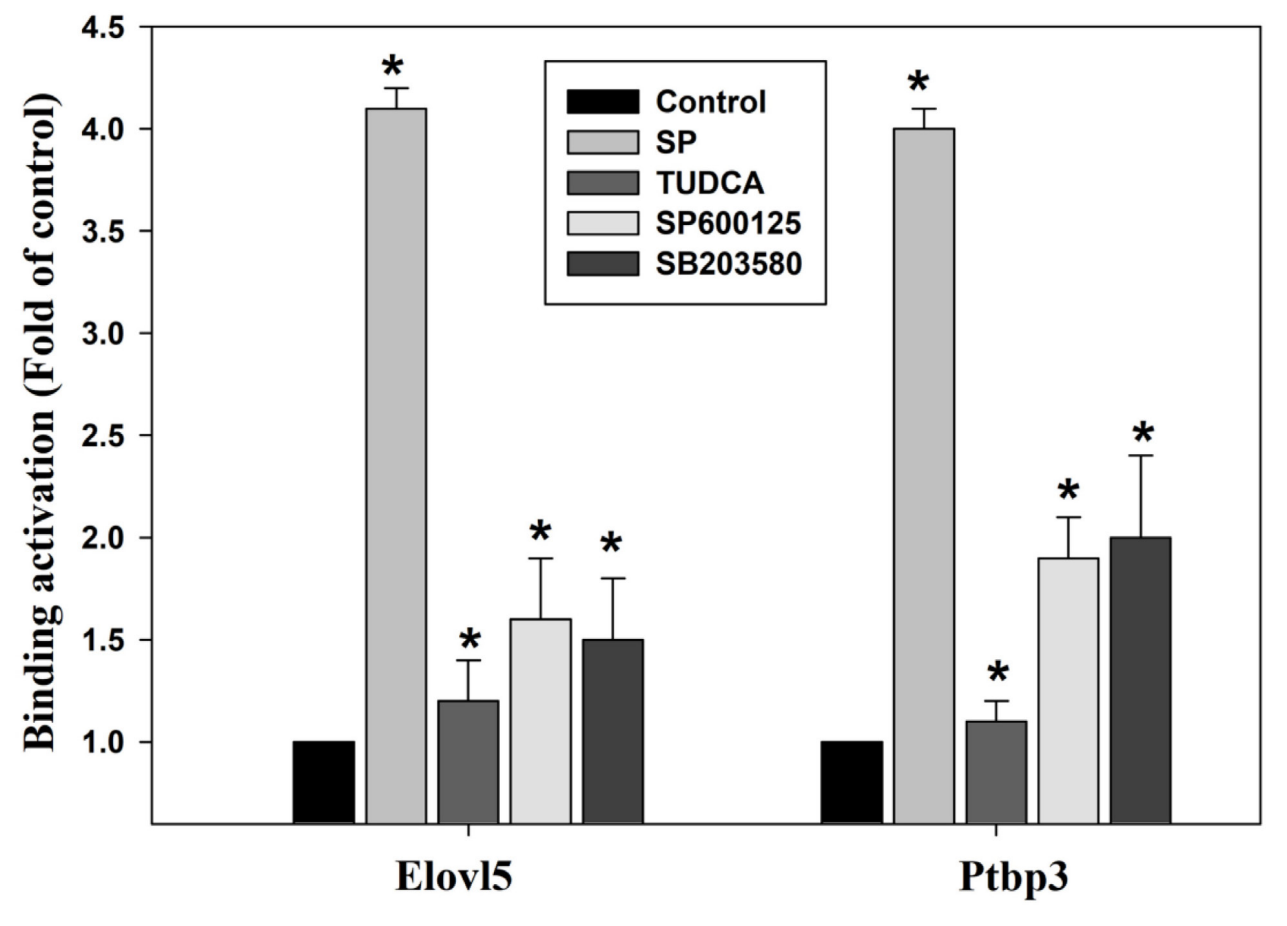
The kinase inhibitors block the binding activities of Elovl5 and Ptbp3 promoter regions, as induced by palmitate treatment. The chromatin immunoprecipitation assay was performed using antibodies against histone Histone H3, trimethylated lysine 4 and Elovl5/Ptbp3 promoters (the -483~-281/-490~-275 target sites, as described in the Materials and Methods) in the precipitated DNA, which was amplified by quantitative real-time polymerase chain reaction using specific primer sets. The liver cells were incubated with or without ER stress inhibitor TUDCA, JNK inhibitor SP600125, or the p38 MAPK inhibitor SB203580 for 24 h at various concentrations. The data are presented as the mean ± SD of three independent experiments. * p < 0.05, as compared to the control group.

The methylation of histones, the H3K4me3 plays an activating effect of the transcriptional activity of genes in the abnormal hepatic lipid metabolism, oxidative stress response, mitochondrial damage and cell apoptosis, particularly in pathogenesis of liver 34,35. We further continued to explore any possible relation of palmitate acid in regulating the H3K4me3 methylated histone modification to induce the increase in the Elovl5/Ptbp3 protein expression by activating the ER stress ASK1/JNK/p38MAPK pathway. The potential effects of the ASK1/JNK/p38MAPK signaling pathway on the epigenetic regulation of histone modifications was also examined. The histone modifications include methylation and acetylation. The nucleic acid modification occurs at the position where the trimethylated lysine 4 of histone H3 is associated with the chromatin. The role of histone methylation in regulating specific genes was identified through ChIP. The results showed that palmitate acid could induce the Elovl5/Ptbp3 gene expression, and facilitate the crucial phenomenon of histone H3K4me3 modification on the promoter binding site (-483~-281/-490~-275) of the genes (Table 4). The effect of the palmitate acid on regulating the H3K4me3 methylated histone modifications was tested using inhibitors. The effects of ER stress-associated proteins ASK1 inhibitor TUDCA (1 mM), JNK inhibitor (50 μM), and p38 inhibitor (20 μM) on Elovl5/Ptbp3 expression and H3K4me3 histone modification varied to different degrees. The ASK1/JNK/p38MAPK pathway affects about 65% respectively. These results showed that palmitate acid can induce the expression of Elovl5/Ptbp3 genes, and increase their expression by regulating the H3K4me3 modification via ASK1/JNK/p38MAPK pathway (Figure 9).

## Discussion

TPN has been widely used in both adult and pediatric patients 1,2. When the gastrointestinal tract of a patient is not functioning properly due to a disease or dysfunction, normal oral intake or adequate nutrient absorption is hindered. To address this, TPN can be used to provide nutritional therapy 3,7. Long-term TPN use can lead to hyperglycemia, cholestasis, steatosis, and liver damage. Excessive levels of nutrition can promote the conversion to triglycerides, which are stored in the liver, which can lead to hepatic steatosis and potentially develop into non-alcoholic fatty liver disease (NAFLD). The dysbalanced nutrient delivery and metabolism during TPN can lead to increased production of ROS, causing oxidative stress 14,36,37. In turn, oxidative stress can contribute to ER stress and cellular damage. To minimize potential complications of TPN, Close monitoring of patients on TPN and individualized nutritional management are essential. Therefore, it is important to seek novel biomarkers for estimating the liver damage of long-term TPN.

This investigation is fundamentally oriented toward establishing an acute liver injury model, rather than investigating the chronic hepatotoxic effects associated with total parenteral nutrition (TPN) 18-21. Acute injury models are distinguished by their compressed temporal frameworks, and in this particular study, the methodology likely represents a metabolic overload paradigm designed to examine the liver's immediate adaptive responses to rapid nutrient influx. Given that rodents exhibit substantially elevated metabolic rates compared to humans, hepatic responses in rats can develop across a considerably abbreviated timeframe. Consequently, pathological or biochemical alterations that would typically necessitate extended exposure periods in human subjects may manifest more expeditiously in the rat model. The investigation therefore concentrates on the acute hepatic effects induced by specific TPN components, including lipid emulsions, amino acids, and high-glucose TPN formulations. While this experimental model does not comprehensively replicate the clinical conditions of extended TPN therapy, it yields valuable mechanistic insights regarding early hepatic responses to parenteral nutrient administration and may potentially elucidate the initial pathophysiological processes that precede chronic liver injury. In our study, male rats (200-250 g) received TPN via the left femoral vein for 3 h at 30 mL/kg/h. A significant inflammation and hepatocyte death were noted in the portal (Z1), periportal (Z2), and terminal hepatic venule (Z3) zones in the TPN group, suggesting that TPN-induced liver injury causes significant functional and histopathological changes. Serological tests also demonstrated increased levels of liver damage markers, with ALT, AST, and ALP levels being 139 ± 5, 84 ± 3, and 641 ± 7 in the TPN group, compared with controls (p<0.05) (as shown in Figure 1, Table 1). In this study, we first used the iTRAQ proteomic approach and identified differentially expressed proteins in samples from model rats for parenteral nutrition-associated liver disease (Figure 2). A total of 6,436 proteins were identified, with 48 yielding quantitative results. Among them, the expression of 19 proteins was significantly increased, while 29 exhibited decreased expression in the TPN group compared to controls (greater than a 1.5-fold difference). Using the PANTHER classification system, these differentially expressed proteins were categorized into 6 KEGG pathways, 17 biological processes, 5 cellular components, and 16 molecular functions. The String database also revealed the interaction network between these proteins, particularly Elovl5 and Ptbp3, further confirming their involvement in lipid metabolism and ER stress (Figure 3, Table 2). The analysis of the 29 proteins whose expression was downregulated identified their role in 10 KEGG pathways, 19 biological processes, 20 cellular components, and 20 molecular functions (as shown in Figure 4, Table 3). The rats' model of parenteral nutrition-associated liver disease was used to study changes in the ER stress markers, Elovl5 and Ptbp3, and their* in vitro* role was determined. Elovl5 and Ptbp3 were markedly involved in ER stress and the TPN-related injury to liver cells 31-33. Immunostaining results further showed that the levels of Elovl5 and Ptbp3 were 2-4 folds higher in the TPN-induced liver injury group compared to those in the control group. Similarly, ER stress-related proteins phospho-IRE1 alpha and phospho-ASK1 also showed an increase in expression in the TPN group. These findings further demonstrate the impact of TPN-induced ER stress on injury to liver cells and highlight the critical roles of Elovl5 and Ptbp3 in the pathological changes observed in liver tissue (Figure 5). Further investigations are warranted to determine if stable Elovl5 shRNA and Ptbp3 shRNA may be useful to minimize TPN-induced liver injury.

The network apparently shows that elongation of very long chain fatty acids protein 5 (Elovl5) and polypyrimidine tract-binding protein 3 (Ptbp3) are linked together, whose levels are affected, focusing specifically on its molecular functions to lipid metabolism by modulating the composition of cellular lipid pools through fatty acid elongation 38. Elovl5 primarily localizes to the ER and plays a role as an enzyme involved in the elongation of fatty acids, particularly as a membrane-bound enzyme involved in lipid metabolism 39. Elevated Elovl5 levels were also observed in the context of ER stress, highlighting its critical role in lipid metabolism and the pathological changes in liver tissue, including lipid accumulation, inflammation, and fibrosis 40. Ptbp3 may be associated with the cellular response to ER stress by regulating the splicing of genes encoding proteins in the UPR pathway to restore ER homeostasis 32,33. Additionally, Ptbp3 can lead to alterations in lipid metabolism, contributing to lipid accumulation in liver cells, a hallmark of NAFLD and non-alcoholic steatohepatitis (NASH), creating a vicious cycle that promotes liver injury. Based on previous reports, our functional studies indicate credible results regarding Elovl5 and Ptbp3. In addition, some novel proteins, which may serve as potential targets for TPN-induced liver injury, were uncovered. In the study, Elovl5 and Ptbp3 expression was knocked-down to evaluate their effects on cell apoptosis and ROS generation (Table 5). Interestingly, Elovl5 and Ptbp3 siRNAs decreased the damage to hepatocytes and the production of ROS potential in hepatocytes. We confirmed these findings *in vitro* by establishing that Elovl5 and Ptbp3 play a role in parenteral nutrition-related liver disease by modulating the ROS generation and cell death. Nonetheless, further study is needed to elaborate the effects of Elovl5 and Ptbp3 in TPN-induced ER stress. The findings will significantly contribute to understanding liver injury through the modulation of key genes involved in the UPR, lipid metabolism, and apoptosis as a potential biomarker or therapeutic target for mitigating liver damage in patients receiving long-term TPN.

TPN has been demonstrated to be involved in ER stress and cell apoptosis, particularly in the liver and intestines, and is associated with factors like nutrient imbalance, lipid overload, and disrupted bile acid metabolism 17,18,41. Prolonged ER stress has been implicated in induced apoptosis through mechanisms such as CHOP induction, caspase activation, calcium dysregulation, and the binding of IRE1α to TRAF2 and ASK1, as well as JNK/p38 pathways 16,42-44. In this study, palmitate acid was shown to induce lipotoxicity in HepG2 and FL83B cells, leading to ROS accumulation and activation of signaling pathways, such as JNK, p38, and ASK1 (Figure 6, Figure 7). Western blotting analysis revealed the generation of free radicals through the ER stress pathway, involving increased expression of Ire1α/TRAF2. Furthermore, the expression of Elovl5 and Ptbp3 contributing to the overall lipotoxic effects of palmitate acid was further investigated. Then, treatment of experimental cells with palmitate acid showed decreased expression of Bcl-2/Bcl-XL and the release of activated caspase-3, caspase-9, and cytochrome C into the cytoplasm (Figure 8A, Figure 8B). Palmitate acid treatment also induced the activation of the ASK1/JNK/p38MAPK pathway within 24 h (Figure 8C). Further experimental studies are required for more specific information regarding the abundances and functional characteristics of Elovl5 and Ptbp3 proteins in hepatocytes during TPN infusion, particularly in the context of fatty acid metabolism and ER stress, to determine the regulatory mechanism involved in liver protection and growth with regards to the Elovl5 and Ptbp3 signaling axis.

Trimethylation of histone H3 at lysine 4 (H3K4me3) is a well-known marker of active gene transcription, often found at promoters of actively transcribed genes 34,45. This modification plays a role in tissue damage and disease progression, particularly in metabolic and liver disorders, by recruiting transcriptional machinery 34. This trimethylation of histone facilitates the binding of transcription factors, RNA polymerase II, and other components crucial for gene transcription. Aberrant H3K4me3 methylation has a complex role in the regulation of hepatocyte genes, particularly in ER stress conditions 41,46. H3K4me3 modification influences critical processes like lipid metabolism and activates pathways such as IRE1α, the activation of caspases, calcium dysregulation, and the ASK1, JNK, and p38 pathways, which promote apoptosis of hepatocytes 47,48. This study examined whether palmitate acid regulates the methylation of histone H3K4, leading to increased expression of Elovl5 and Ptbp3 through the ER stress/JNK/p38MAPK pathway. ChIP analysis revealed that palmitate acid could induce Elovl5 and Ptbp3 expression and modify H3K4me3 at specific DNA binding sites on their promoters. This study additionally demonstrated that palmitate acid modulates Elovl5/Ptbp3 expression and H3K4me3 methylation through the ER stress ASK1/JNK/p38MAPK pathway (Figure 9). Finally, our findings suggest that Elovl5 and Ptbp3 might serve as novel regulators and potential biomarkers or therapeutic targets to improve liver damage in patients on long-term TPN, as their expression is regulated by the ASK1/JNK/p38MAPK pathway and H3K4 methylation.

## Conclusions

**Figure 10:**
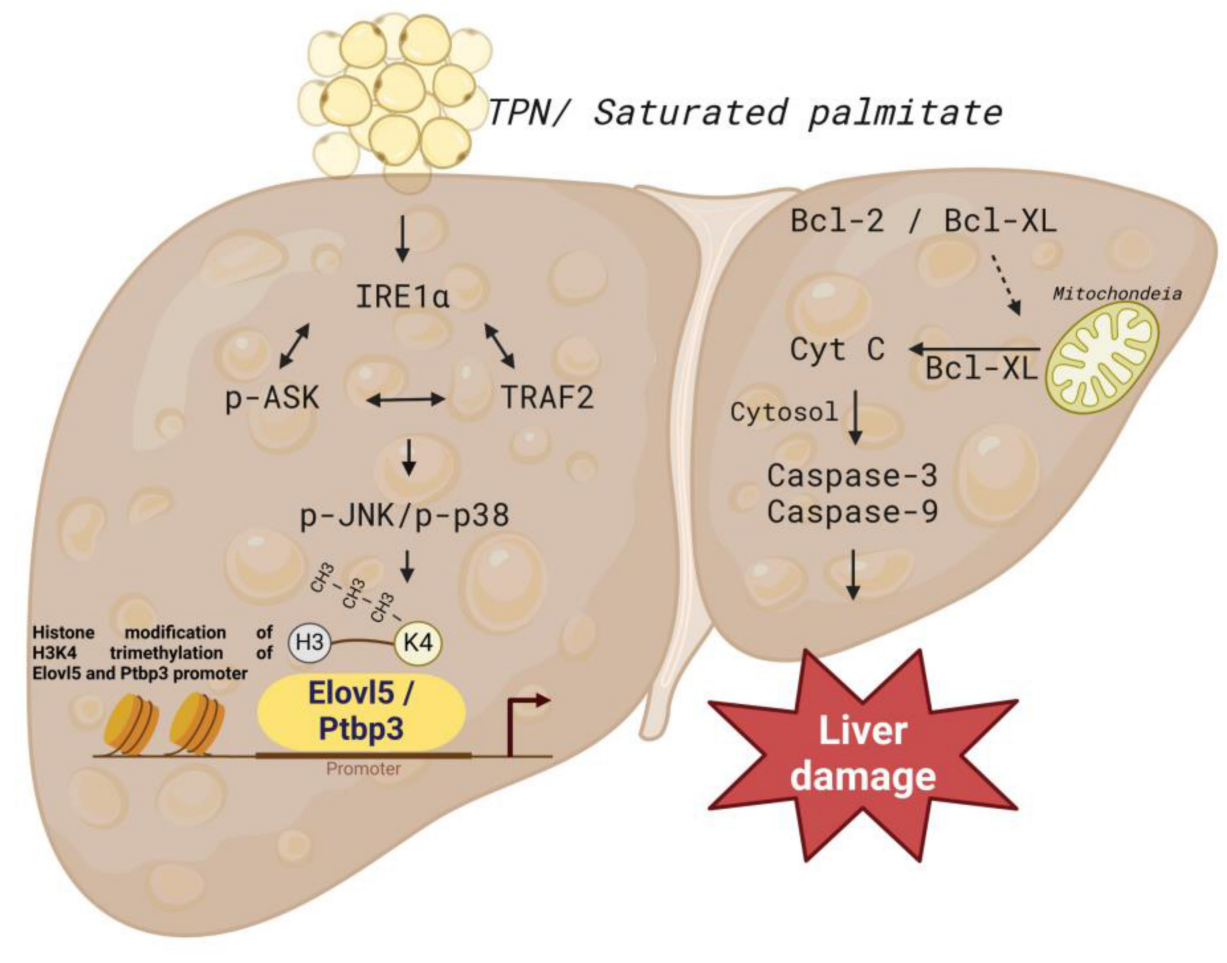
Schematic presentation of the molecular mechanism of palmitate -mediated apoptotic induction and endoplasmic reticulum stress of liver cells. Sodium palmitate treatment increases the expression of Elovl5 and Ptbp3 through ER stress-derived and ASK1/JNK/p38 MAPK-signaling-pathway-mediated histone H3K4me3. Activation of Elovl5 and Ptbp3 by palmitate triggers liver cells death through the inhibition of Bcl-2 and Bcl-XL, activated caspase-3, and -9.

In this study, the effects of TPN on rat liver cells was comprehensively explored, and the proteomics methods were applied to analyze the profiles of differentially expressed proteins in animal experiments. We particularly focused on the functions of Elovl5 and Ptbp3 proteins in the process of liver injury. The findings revealed that in hepatocytes and animal models treated with TPN, the expression of Elovl5 and Ptbp3 proteins was closely related to the degree of hepatocyte inflammation. These proteins potentially have an important role in the process of liver injury, especially in the mechanism of response to oxidative damage. The palmitate acid can induce liver cell apoptosis and lead to the generation of free radicals; this process is implemented through the ER stress signaling pathway (e.g., p-IRE1α/TRAF2/ASK1/JNK/p38) and the regulation of Elovl5 and Ptbp3 expressions (Figure 10). In addition, the palmitate can induce the expression of genes coding for Elovl5 and Ptbp3, through a process related to the regulation of H3K4me3 histone modification. The activation of Elovl5 and Ptbp3 was demonstrated to further inhibit the expression of anti-apoptotic proteins Bcl-2 and Bcl-XL and activate caspase-3 and caspase-9 expressions, ultimately leading to liver cell apoptosis.

## Figures and Tables

**Table 1:** Mean serum liver function in total parenteral nutrition rats

Group	Normal	TPN
GOT (IU/L)	40 ± 5	139 ± 5
GPT (IU/L)	25 ± 4	84 ± 3
ALP (IU/L)	544 ± 8	641 ± 7

Normal: Normal saline; TPN: Kabiven; TPN Flow: 30mL / kg B.W. / hr × 3hr; GOT: Aspartate Aminotransferase; GPT: Alanine Aminotransferase; ALP: Alkaline Phosphatase.

**Table 2:** Proteins that are up regulated in TPN by iTRAQ analysis

No.	Accession	Gene symbol	Name	MW [kDa]	Ratio: TPN / N	Ratio: TPN / N
1	P05182	Cyp2e1	Cytochrome P450 2E1	56.6	1.75	1.73
2	P00689	Amy2	Pancreatic alpha-amylase	57.1	1.79	1.92
3	Q5M828	Adtrp	Androgen-dependent TFPI-regulating protein	26.9	1.96	1.87
4	Q6AY80	Nqo2	Ribosyldihydronicotinamide dehydrogenase	26.3	2.03	1.71
5	Q6PCU8	Ndufv3	NADH dehydrogenase [ubiquinone] flavoprotein 3, mitochondrial	11.9	2.08	1.69
6	Q920L7	Elovl5	Elongation of very long chain fatty acids protein 5	35.2	2.16	2.00
7	P50169	Rdh3	Retinol dehydrogenase 3	35.6	5.95	6.54
8	Q64581	Cyp3a18	Cytochrome P450 3A18	57.3	1.45	1.47
9	P51590	Cyp2j3	Cytochrome P450 2J3	57.9	1.45	1.55
10	Q9Z118	Ptbp3	Polypyrimidine tract-binding protein 3	56.7	1.47	1.60
11	P05183	Cyp3a2	Cytochrome P450 3A2	57.7	1.48	1.51
12	P11711	Cyp2a1	Cytochrome P450 2A1	56	1.50	1.44
13	P31646	Slc6a13	Sodium- and chloride-dependent GABA transporter 2	68.2	1.51	1.47
14	Q9JKC9	Synrg	Synergin gamma	141.3	1.53	1.45
15	Q5BJN5	Chchd4	Mitochondrial intermembrane space import and assembly protein 40	15.5	1.57	1.44
16	P49889	Ste	Estrogen sulfotransferase, isoform 3	35.4	1.62	3.47
17	P13255	Gnmt	Glycine N-methyltransferase	32.5	1.62	1.49
18	P15149	Cyp2a2	Cytochrome P450 2A2	56.3	1.65	1.43
19	Q6AXR5	Slc35a3	UDP-N-acetylglucosamine transporter	36.1	1.65	1.48

**Table 3:** Proteins that are down regulated in TPN by iTRAQ analysis

No.	Accession	Gene symbol	Name	MW [kDa]	Ratio: TPN / N	Ratio: TPN / N
1	Q5M8C6	Fgl1	Fibrinogen-like protein 1	36.5	0.40	0.49
2	P02764	Orm1	Alpha-1-acid glycoprotein	23.6	0.41	0.43
3	P11510	Cyp2c12	Cytochrome P450 2C12, female-specific	55.9	0.43	0.41
4	Q4G075	Serpinb1a	Leukocyte elastase inhibitor A	42.7	0.43	0.60
5	P08932		T-kininogen 2	47.7	0.47	0.52
6	Q62730	Hsd17b2	Estradiol 17-beta-dehydrogenase 2	41.9	0.48	0.52
7	P06866	Hp	Haptoglobin	38.5	0.48	0.65
8	P09006	Serpina3n	Serine protease inhibitor A3N	46.6	0.48	0.61
9	P34901	Sdc4	Syndecan-4	21.9	0.48	0.55
10	P40190	Il6st	Interleukin-6 receptor subunit beta	102.4	0.48	0.56
11	Q65Z14	Osmr	Oncostatin-M-specific receptor subunit beta	108.6	0.49	0.51
12	P30152	Lcn2	Neutrophil gelatinase-associated lipocalin	22.5	0.49	0.42
13	Q5QE78	Aox2	Aldehyde oxidase 2	147.8	0.51	0.65
14	O54980	Syngr2	Synaptogyrin-2	24.7	0.51	0.54
15	P25086	Il1rn	Interleukin-1 receptor antagonist protein	20.3	0.52	0.52
16	P12839	Nefm	Neurofilament medium polypeptide	95.7	0.53	0.53
17	O08560	Dgkz	Diacylglycerol kinase zeta	103.9	0.54	0.65
18	Q5RJT2	Ftsj3	pre-rRNA processing protein FTSJ3	94.7	0.54	0.62
19	P36201	Crip2	Cysteine-rich protein 2	22.7	0.59	0.61
20	Q9EQS5	Sc5d	Lathosterol oxidase	35.1	0.61	0.48
21	Q66H85	Ankzf1	Ankyrin repeat and zinc finger domain-containing protein 1	80.5	0.62	0.60
22	P35446	Spon1	Spondin-1	90.7	0.62	0.65
23	Q6P6U0	Fgr	Tyrosine-protein kinase Fgr	58.8	0.64	0.59
24	Q9EPJ0	Nucks1	Nuclear ubiquitous casein and cyclin-dependent kinase substrate 1	27.1	0.64	0.60
25	P18265	Gsk3a	Glycogen synthase kinase-3 alpha	51	0.65	0.64
26	Q6AYL5	Sf3b4	Splicing factor 3B subunit 4	44.3	0.65	0.59
27	Q63189	Prg2	Bone marrow proteoglycan =	25.1	0.66	0.60
28	P27791	Prkaca	cAMP-dependent protein kinase catalytic subunit alpha	40.6	0.66	0.53
29	P23928	Cryab	Alpha-crystallin B chain	20.1	0.67	0.36

**Table 4:** The specific primers.

Ptbp3 -490 to -275 bp
Ptbp3 F 5'- TGCTCTCAGGTCCGGAAAG -3'
Ptbp3 R 5'- CGACTCGCCCTCCTACAAG-3'
Elovl5 -483 to -281 bp
Elovl5 F 5'- TGCTTCAATTTGCGCAACAG-3'
Elovl5 R 5'- AGTGAAGTTCTGGATGCCCT-3'

**Table 5:** The effects of Elongation of very long chain fatty acids protein 5 (Elovl5) and Polypyrimidine tract-binding protein 3 (Ptbp3) inactivation on cell apoptosis and ROS of palmitate treatment in hepatocytes.

	Apoptosis (%)	ROS generation (Fold)
Control	8 ± 2	1
Sodium palmitate	20 ± 3	3.0 ± 0.5
Sodium palmitate + siRNA Elovl5	13 ± 2	1.8 ± 2
Sodium palmitate + siRNA Ptbp3	11 ±2	2.0 ± 2

## Abbreviations

ASK1: Apoptosis Signal-regulating Kinase 1
CHOP: C/EBP Homologous Protein
Elovl5: Elongation of Very Long Chain Fatty Acids Protein 5
ER stress: Endoplasmic Reticulum Stress
IHC: Immunohistochemistry Stain
IRE1α: Inositol-requiring Enzyme Type 1
iTRAQ: Isobaric Tags for Relative and Absolute Quantitation
JNK: Jun N-terminal Kinase
Ptbp3: Polypyrimidine Tract-binding Protein 3
ROS: Reactive Oxygen Species
TRAF2: Receptor-associated Factor 2
TPN: Total Parenteral Nutrition
TNF: Tumor Necrosis Factor
UPR: Unfolded Protein Response
